# Initiator Elements Function to Determine the Activity State of BX-C Enhancers

**DOI:** 10.1371/journal.pgen.1001260

**Published:** 2010-12-23

**Authors:** Carole Iampietro, Maheshwar Gummalla, Annick Mutero, François Karch, Robert K. Maeda

**Affiliations:** National Centre of Competence in Research (NCCR) Frontiers in Genetics and Department of Zoology and Animal Biology, University of Geneva, Geneva, Switzerland; Harvard Medical School, Howard Hughes Medical Institute, United States of America

## Abstract

A >300 kb *cis*-regulatory region is required for the proper expression of the three bithorax complex (BX-C) homeotic genes. Based on genetic and transgenic analysis, a model has been proposed in which the numerous BX-C *cis*-regulatory elements are spatially restricted through the activation or repression of parasegment-specific chromatin domains. Particular early embryonic enhancers, called initiators, have been proposed to control this complex process. Here, in order to better understand the process of domain activation, we have undertaken a systematic *in situ* dissection of the *iab-6 cis*-regulatory domain using a new method, called InSIRT. Using this method, we create and genetically characterize mutations affecting iab-6 function, including mutations specifically modifying the *iab*-6 initiator. Through our mutagenesis of the *iab*-6 initiator, we provide strong evidence that initiators function not to directly control homeotic gene expression but rather as domain control centers to determine the activity state of the enhancers and silencers within a *cis*-regulatory domain.

## Author Summary

Understanding how genes become activated is one of the primary areas of research in modern biology. In order to decipher the DNA components required for this process, scientists have traditionally turned to transgenic reporter assays, where DNA elements are removed from their native environment and placed next to a simplified reporter gene to monitor transcriptional activation. Although this approach is powerful, it can result in artifacts stemming from the channelization of regulatory element activities into predetermined classes. In this manuscript, we investigate the biological role of elements from the *Drosophila* bithorax complex, called initiators. In transgenic assays, these elements have been categorized as enhancers. However, genetic analysis suggests that, *in situ*, these elements perform a far more complex function. Here, using a new method to repeatedly target a genetic locus for mutagenesis, we show that initiators function as control elements that coordinate the activity of nearby enhancers and silencers. Overall, our study highlights how gene expression can be controlled through a hierarchical arrangement of *cis*-regulatory elements.

## Introduction

The *Drosophila* bithorax complex (BX-C) is one of two homeotic gene clusters in the fly and is responsible for determining the segmental identity of the posterior thoracic segment and each of the fly abdominal segments [1], [2]. It does this by using a >300 kb *cis*-regulatory region to control the parasegement-specific expression of the three BX-C homeotic genes: *Ubx, abd-A and Abd-B* (for review, see [3]).

Through the early genetic analysis of the BX-C, it was shown that its *cis*-regulatory sequences can be divided into nine parasegment-specific chromosomal domains (*abx/bx, bxd/pbx, and iab-2* through *iab-8*), where each domain controls the activation of one of the three BX-C homeotic genes in a pattern appropriate for that parasegment [4]–[8]. Since their identification, these domains have been dissected using transgenic reporter assays to identify individual regulatory elements capable of modifying reporter gene expression. Among the elements identified were early embryonic enhancers (initiators), cell-type-specific enhancers, silencers and insulators [9]–[22]. Interestingly, although homeotic gene expression is restricted along the antero-postero (A–P) axis, many of the elements identified by transgenic analysis do not control reporter gene expression in an A–P restricted manner. These findings, when combined with the early genetic data suggest a model in which the *cis*-regulatory elements of the BX-C are controlled through the activation or repression of parasegment-specific chromatin domains [4], [23]–[24].

According to this model, the BX-C functions through multiple layers of control. First, there are the enhancers that directly activate homeotic gene expression in a pattern appropriate for a specific parasegment. Based on the genetic data, these enhancers are known to be grouped in a way where all the enhancers required to produce a PS-specific pattern of homeotic gene expression are clustered into domains within the BX-C sequence. However, although these enhancers produce a pattern of homeotic gene expression appropriate for a specific parasegment, in transgenic assays, they are not restricted along the A–P axis, and are only restricted to specific cell-types [9], [12], [22], [25].

The second layer of control comes from Polycomb-response element silencers (PREs). These silencers are thought to turn off the clusters of enhancers in parasegments where they are not needed, via modification of the local chromatin structure around the enhancers (for reviews [26]–[28]). Once again, however, like the cell-type-specific enhancers, by themselves, PREs do not seem to sense positional information and can silence genes regardless of A–P position [29].

Domain boundary elements form a third layer of BX-C control. Each of the PS-specific enhancer clusters seems to be flanked by boundary elements, required to keep each cluster separate and autonomous from other clusters. *In situ*, loss of a domain boundary causes the fusion of PS-specific domains, resulting in mutant phenotypes, where the affected segments displays phenotypes characteristic of the other [18], [22], [30]–[31] (for review see [32]). In transgenic assays, these elements have been shown to behave as insulators, blocking both positive and negative effects of *cis*-regulatory elements on reporter gene activity [13]–[14], [17]–[18], [21]. However, the presence of boundary elements cannot explain the A–P restriction of the BX-C regulatory elements, for, as with the enhancers and silencers, when taken out of the BX-C, boundary elements do not seem to have an A–P restricted activity.

So then, how do non-restricted regulatory elements control homeotic gene expression in an A–P position-dependent manner? If we are to assume that the reporter gene assays represent a reasonable estimate of the activity of the various regulatory elements in a domain, then there must be some other element in each domain that coordinates the activity of these elements across the A–P axis. Special early embryonic enhancers, called initiators, are the prime candidates to perform this function [9]–[12], [17]–[18] [20], [22]. As mentioned above, the activity of most of the elements isolated from the BX-C is not restricted along the A-P axis. In this respect, however, initiator elements are exceptional. In transgenic assays, these elements behave as early embryonic enhancers that activate reporter gene expression in a pattern along the A-P axis, consistent with the activity of the domain from which it was isolated. For example, the initiator identified from the *iab-5* domain, which controls *Abd-B* expression in PS10/A5, activates reporter gene expression in PS10/A5 and more posterior segments in a pair-rule fashion [12]. Because similar elements were found in many PS-specific domains and these elements were the only elements discovered in the BX-C capable of reading the early parasegmental address set up by the maternal, gap and pair-rule gene products, it was hypothesized that initiators would act as the primary switches to determine if a domain was active or silenced. Unfortunately, although initiators are thought to play such a key role in BX-C gene regulation, their actual role has never been directly tested *in vivo* due to the lack of appropriate mutations and the difficulties in performing homologous recombination in *Drosophila.*

**Figure 1:**
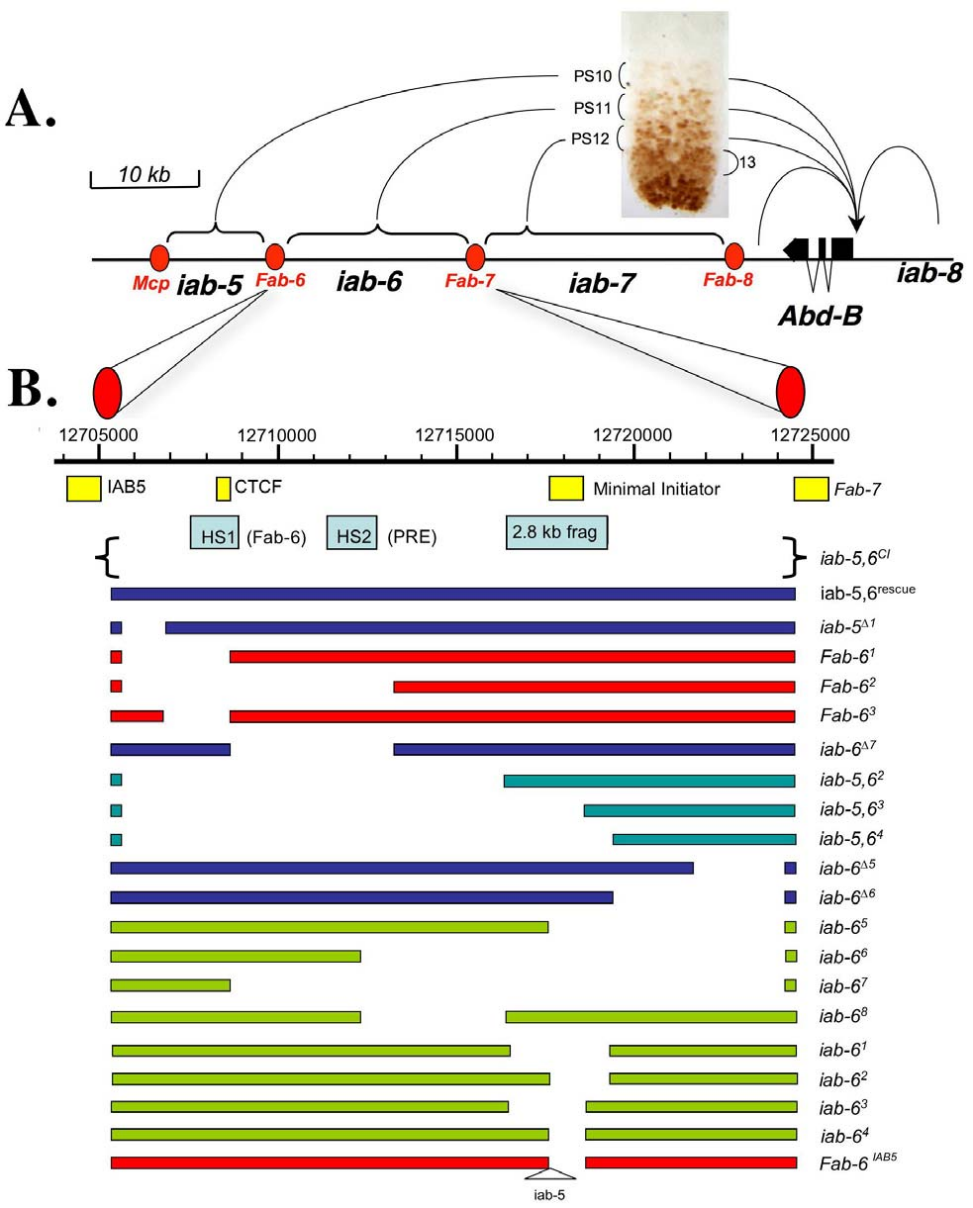
Synopsis of the *Abd-B* locus of the BX-C and diagram of the mutations created for this study. A. Synopsis of the *Abd-B* locus of the BX-C. Diagram of the *Abd-B* gene and its 3′*cis-*regulatory region. The horizontal line represents the DNA sequence of the BX-C (see scale on top left). The *Abd-B* expression pattern in the central nervous system of a 10 hours embryo is shown above the DNA line. In parasegment 10 (PS10) *Abd-B* is present in a few nuclei at a relatively low level. This PS10-specific expression pattern is controlled by the *iab-5* regulatory domain located 55 kb downstream from the *Abd-B* promoter. In PS11, PS12 and PS13, *Abd-B* is present in progressively more nuclei and at higher levels. These patterns are controlled by the *iab-6*, *iab-7* and *iab-8* regulatory domains, respectively. Each regulatory domain functions autonomously from its neighbors due to the presence of the boundaries that flank them (red ovals). B. Diagram of the mutations created for this study. The top line shows the DNA coordinates of *iab-6*, according to the *Drosophila* Genome Project. Below this line, and to approximate scale, are the locations of the various elements isolated from the BX-C including the IAB5 initiator[12], DNase hypersentive site 1 (HS1/*Fab-6* including the CTCF binding sites) and 2 (HS2/PRE) [43]–[44], the 2.8 kb *iab-6* initiator fragment [22], the minimal initiator fragment and the *Fab-7* boundary [14], [30]. Below this line are the DNAs reintegrated to make the mutations. The various *iab-6* alleles are indicated as solid bars, with gaps indicating the areas deleted. These bars are color coded such that blue bars indicate mutants that show no cuticle or CNS phenotypes at 25°C, red bars indicate mutants with *Fab-6*-type phenotypes, turquoise bars indicate mutants with *iab-5,6* phenotypes, and green bars indicate mutants with *iab-6* phenotypes.

Thus, in order to explore the function of initiators and other regulatory elements *in vivo*, we developed a method to streamline the homologous recombination process for rapid, precise, and systematic mutagenesis. Using this method, called InSIRT (In Situ Integration for Repeated Targeting), we have created twenty new mutations in the *iab-6* region of the BX-C, including mutations that directly test the role of the initiator in BX-C gene regulation (Figure 1 and Table 1).

**Table 1:** Mutations and phenotypes.

Mutation	Region Deleted	A5	A6	Oligos
*iab-5,6^CI^*	3R:12705250–12724572	A4/A5	A4/A5	
*iab-5^Δ1^*	*3R:12705410–12706585*	A5	A6	D1+P7
*Fab-6^1^*	*3R:12705410–12708661*	A4/A6	A4–5/A6	D1+P6
*Fab-6^2^*	*3R: 12705410–12713431*	A4/A6	A4–5/A6	D1+P5
*Fab-6^3^*	*3R:12706583–12708661*	A4/A6	A4–5/A6	D2+P6
*Fab-6^4+IAB5^*	*3R:12717635–12718561†*	A6	A6	D6+P3
*iab-6^1^*	*3R:12716494–12719281*	A5	A5	D5+P2
*iab-6^2^*	*3R:12717635–12719281*	A5	A5	D6+P2
*iab-6^3^*	*3R:12716494–12718561*	A5	A5	D5+P3
*iab-6^4^*	*3R:12717635–12718561*	A5	A5	D6+P3
*iab-6^5^*	*3R:12717635–12724398*	A5	A5	D6+P1
*iab-6^6^*	*3R:12712171–12724398*	A5*	A5*	D4+P1
*iab-6^7^*	*3R:12708714–12724398*	A5*	A5*	D3+P1
*iab-6^8^*	*3R:12712171–12716492*	A5*	A5/A6	D4+P4
*iab-6^Δ5^*	*3R:12721546–12724398*	A5	A6**	D8+P1
*iab-6^Δ6^*	*3R:12719282–12724398*	A5	A6**	D7+P1
*iab-6^Δ7^*	*3R:12708714–12713431*	A5	A6	D3+P5
*iab-5,6^2^*	*3R:12705410–12716492*	A4/A6	A4/A6	D1+P4
*iab-5,6^3^*	*3R:12705410–12718561*	A4/A5	A4/A5	D1+P3
*iab-5,6^4^*	*3R:12705410–12719281*	A4/A5	A4/A5	D1+P2

Column one lists the name of each mutation created for this work. Column two indicates the regions deleted in each line based on the coordinates of the Drosophila genome project (Release 5). Columns three and four summarize the transformations observed in the cuticle and embryonic CNS of A5/PS10 (labeled A5) and A6/PS11 (labeled A6). (†) indicates that in the *Fab-6^4+IAB5^* mutation, the sequence indicated was deleted, but was replaced by the sequence of the IAB5 initiator. (*) has been used to indicate that the cuticle trichome pattern occasionally shows signs of weak transformation towards A6. Based on other observations, we believe this could be a *cis-*over expression effect [29], [57] due to the loss of PRE sequences. (**) Mutations *iab-6^Δ5^* and *iab-6^Δ6^* display a slight loss of A6 cuticle at 18°C. Finally column five indicates the oligos listed in Table 2 that were used to generate the corresponding deficiencies.

**Table 2:** Oligos used to generate the deletions.

Sequence	Dist
TGCCACTCACGCAGGACCCAGTTCCATCGAGGGATATTTATAGGGAGATG**GAATACAAGCTTGGGCTGCAGG**	D5
CAGAAATGTAAAATAAACCTTTAATATTTTCTAACCATTCCAGAAATCTG**GAATACAAGCTTGGGCTGCAGG**	D6
TACGAGCGAGCATCTTGCCAAAATGAGAAAACTTTTGCCAACACCAAACG**GAATACAAGCTTGGGCTGCAGG**	D1
GACTAAACTCGAAGCACTTGAGCCGGCATATCTTTTTAATTTGGACGAGG**GAATACAAGCTTGGGCTGCAGG**	D2
CCGACGACCGAGTGGTGGAATACGCGGAATCTGGTTAACAATCTTGTTTG**GAATACAAGCTTGGGCTGCAGG**	D3
AAGTGCCCACTGTGCGCATGTGCGGGATTTCGCGTTGCCACGACCCATGG**GAATACAAGCTTGGGCTGCAGG**	D7
CGAGGGTCACAAAAAGAGGGGGCGGGGTGCTGGTTCCATGTGTCCCAGCC**GAATACAAGCTTGGGCTGCAGG**	D4
GGCAGCACGAATAGTTTAGTTTATTTTAGCCATAGCTCAAGAACGACAGC**GAATACAAGCTTGGGCTGCAGG**	D8
	Prox
TGGCGTCATGTACCAGAATTTTCTTTGGCGGTGGAAAAGCGAGCAATTTC**CTCGCCCGGGGATCCTCTAGAG**	P2
GTCACTCGTTTTTCCAGTAATAAGGAGTATAAAATATATTAACTTACTGC**CTCGCCCGGGGATCCTCTAGAG**	P3
AACAAGATTGTTAACCAGATTCCGCGTATTCCACCACTCGGTCGTCGGCC**CTCGCCCGGGGATCCTCTAGAG**	P6
GCACGAAAGACCCACAACTGGACCCCGTGGAATATGAATGCATCTCGAGC**CTCGCCCGGGGATCCTCTAGAG**	P4
TTGGCAACAAAGTTGGATGCATTGTGGGTGGCAAAATATCAAACAATGGC**CTCGCCCGGGGATCCTCTAGAG**	P1
CTTGGGCGAAGGGTTCGGCACTGGCTTCATTAAGTGCCAGAAGGTGCTGC**CTCGCCCGGGGATCCTCTAGAG**	P5
TGCTGGGGATATAAAAGAAAAGTTTGGCAGGCCAAAATATTGGCCAACAC**CTCGCCCGGGGATCCTCTAGAG**	P7

Bold sequences correspond to the FRT-kanamycin-FRT sequences used to prime the amplification of the FRT-kanamycin-FRT cassette. Regular characters correspond to the homology regions used to generate the deletions by recombineering. P1–P7 correspond to the oligos used to generate the proximal breakpoint of the deletions (relative to the *Abd-B* promoter), while D1–D8 correspond to the oligos used to generate the distal breakpoint.

## Results/Discussion

### Creation of an *attP* integration site in the BX-C

**Figure 2:**
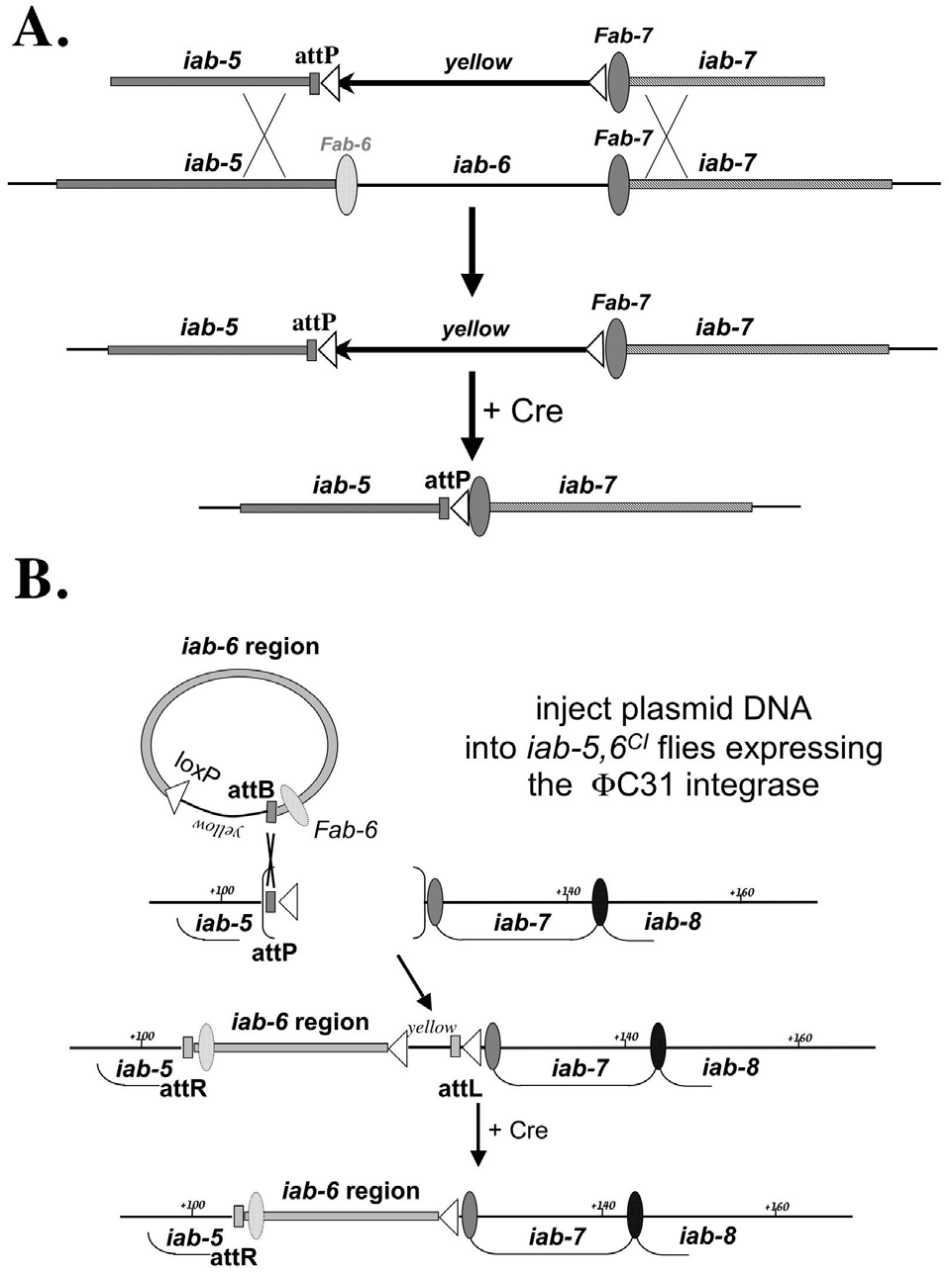
InSIRT. A. Step one: Homologous Recombination. The original “ends-out” donor vector (pW25) was modified to contain an *attP* insertion site and a removable *yellow* reporter gene. Using the yellow reporter, homologous recombination events could be identified by screening for flies with yellow expression in the A5 and A6 segments (a consequence of having *yellow* inserted in the *iab-5* domain). The yellow reporter could then be removed to leave only the *attP* site and a single *loxP* recombination site (white triangle) in place of *iab-6*. B. Step two: Reintegration. Plasmids containing a 288 bp *attB* site, a single *loxP* site, a yellow reporter and a version of the 19.3 kb fragment were injected into *iab-5,6^CI^* embryos expressing a maternally supplied φC31 integrase [36]. Integration events were isolated based on yellow gene expression, then crossed to the Cre recombinase to remove the yellow gene and all vector backbone sequence.

To study the *cis-*regulatory elements regulating BX-C homeotic gene expression within their natural chromosomal environment, we sought to design a method that could be used to rapidly and repeatedly target the BX-C for site-specific mutagenesis. Because this method is related to the SIRT method [33], we named it InSIRT for In Situ Integration for Repeated Targeting. Figure 2 provides a rough schematic of this method as used here in the BX-C. In short, homologous recombination is used to replace a genomic region of interest with an entry site (*attP*) recognized by the φC31-bacteriophage integrase [34]–[36]. Once a region of the genome is replaced by an *attP* site, a DNA fragment corresponding to the deleted region can be systematically mutagenized *in vitro* and reinserted into its normal chromosomal location by φC31 integration. As φC31 integration is a relatively fast process (by genetic standards), InSIRT allows site-specific mutagenesis of actual genes to be accomplished within the timeframe required to create a simple transgenic fly. For our experiments, we replaced a 19.3 kb region of the BX-C, roughly corresponding to the *iab-6 cis*-regulatory domain, with a 255bp φC31 integrase *attP* site (Figure 1 and Figure 2; note that the previously identified IAB5 initiator fragment [12] and the *Fab-7* boundary element [30]–[31] are left intact by the deletion, while the area presumed to be the *Fab-6* boundary is removed [22]).

**Figure 3:**
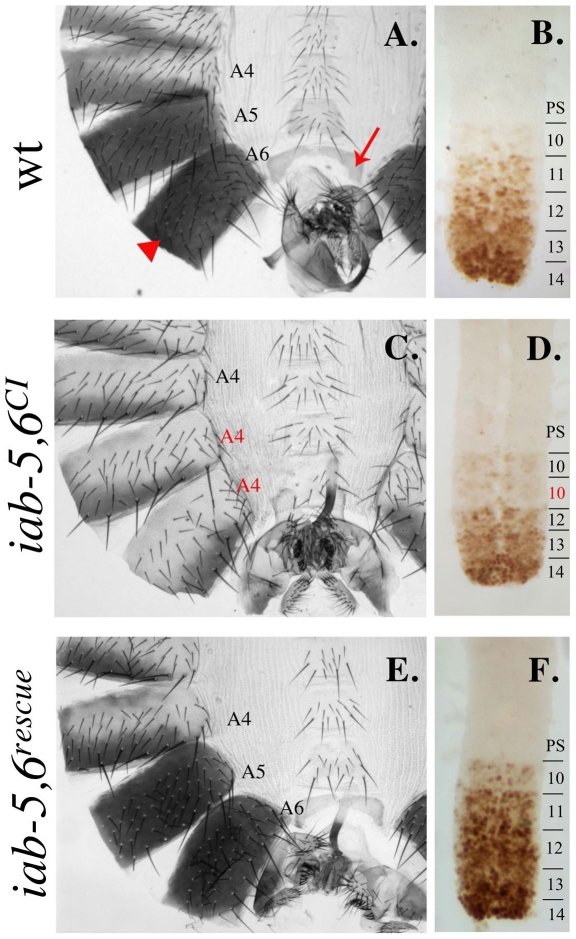
*iab-5,6^CI^* phenotype and rescue. A. A wild-type adult male cuticle with A4-A6 labeled. Segment A5 differs from A6 based on the sternite shape and the bristles present on the A5 sternite. For reference, the A6 tergite is indicated by a red arrowhead and the A6 sternite is indicated by a red arrow. B. A wild-type embryonic nerve cord (anterior towards the top) stained with an antibody to *Abd-B* (brown). Notice the step gradient of *Abd-B* expression increasing in each parasegment towards the posterior. C. An adult male cuticle of a fly homozygous for the *iab-5,6^CI^* chromosome with A5 and A6 transformed towards A4 (notice the A4-like pigmentation on the tergites and the bristled sternites). D. The embryonic nerve cord of homozygous *iab-5,6^CI^* embryos shows only a transformation of A6 into A5, as seen by the repetition of PS10/A5-like Abd-B levels in PS11/A6, indicating that the inactivation of *iab-5* is incomplete and not seen in the embryo. E. An adult male cuticle from a fly homozygous for the *iab-5,6^rescue^* chromosome, where the entire 19.3 kb area deleted in *iab-5,6^CI^* is reintegrated into *iab-5,6^CI^*, looks completely wild type. F. The complete rescue is confirmed by the wild-type pattern of *Abd-B* in the embryonic ventral nerve cord.

Removal of *cis*-regulatory domains in the BX-C typically results in the homeotic transformations of posterior segments towards anterior segments. The segments transformed depend on the *cis*-regulatory domains removed and are transformed towards the last more-anterior segment whose *cis*-regulatory domain is intact. For example, deletion of the *iab-6 cis*-regulatory domain should result in the transformation of segment A6 (whose development in specified by *iab-6*) into A5 (whose development is controlled by *iab-5*). As we were attempting to delete only *iab-6* in our deletion, we thus expected flies homozygous for our 19.3 kb deletion to display a typical iab-6 mutant phenotype. However, this was not the case. Flies homozygous for our 19.3 kb deletion have both their A5 and A6 segments transformed towards A4, indicating that both *iab-5* and *iab-6* activity are affected by our deletion (Figure 3). This can be clearly seen on the adult cuticle. Most of the segments of the adult fly abdomen can be identified independent of their position, by distinct cuticular features. For example, the wild type male sixth segment (A6) is distinguished from other segments by having a darkly pigmented tergite, covered in a distinctive pattern of tiny hairs, called trichomes, and is devoid of sternite bristles. Meanwhile, the fifth segment (A5) displays a similar darkly pigmented tergite that is uniformly covered with trichomes, and has a sternite with numerous bristles (Figure 3A). Flies homozygous for our deletion display an A4-like pigmentation pattern on both the male fifth and sixth abdominal tergites (Figure 3C). Also, the A6 sternite, normally devoid of bristles, displays numerous bristle like the A4 or A5 sternite. Based on these phenotypes, we named our deletion *iab-5,6^CI^*.

Although the adult cuticular phenotypes indicate that *iab-5* function is affected in *iab-5,6^CI^*, this inactivation is incomplete and only some PS10/A5 phenotypes are affected. For example, in the embryonic CNS, the PS10/A5 *Abd-B* expression pattern is normal, indicating that in embryos, *iab-5* is still active (compare Figure 3B with Figure 3D). Also, while *iab-5* null mutants are sterile, *iab-5,6^CI^* mutants are fertile. Based on these results, we believe that *iab-5,6^CI^* removes an adult cuticle enhancer from *iab-5*, while leaving the rest of the *iab-5* cis-regulatory domain intact. *iab-6* function, as would be expected of the deletion we created, seems to be universally affected, as both the adult cuticle, and the embryonic CNS staining are affected (Figure 3C, 3D).

As a control for the InSIRT method, we first decided to reintegrate the 19.3 kb fragment removed in *iab-5,6^CI^*. As expected, the reintegrated line, *iab-5,6 ^rescue^*, reverts all phenotypes associated with *iab-5,6^CI^* and demonstrates the feasibility of our approach (Figure 3E, 3F).

To begin our dissection of the *cis-*regulatory elements in the *iab-6* domain, we created a series of overlapping deletions spanning the 19.3 kb *iab-5,6^CI^* region (Figure 1 and Table 1) and examined their resulting phenotypes on the adult cuticle and embryonic CNS. We will first discuss deletions affecting the *iab-6* initiator.

### The *iab-6* initiator is necessary, but not sufficient to control *Abd-B* expression in PS11/A6

**Figure 4:**
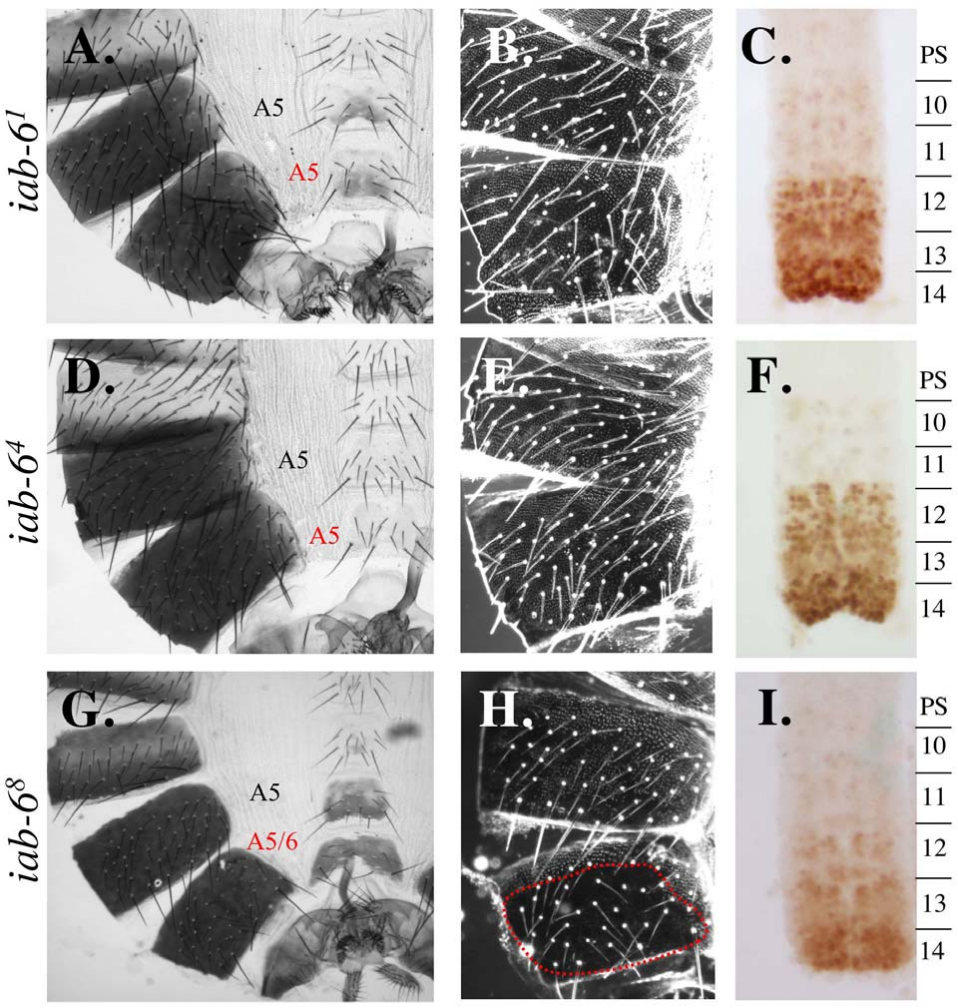
Phenotypes from initiator mutants. Genotypes are as follows: A.–C. *iab-6^1^*, D.–F. *iab-6^4^* and G.–I. *iab-6^8^*. A., D. and G. show adult male cuticles. B., E. and H. show pseudo-darkfield views of the fifth and sixth tergites to visualize the trichome patterns. C., F. and I. show the Abd-B staining pattern in the embryonic nerve cord. In wild-type flies, A5/PS10 differs from A6/PS11 based on the sternite shape, the bristles present on the A5 sternite, the trichome pattern on the fifth and sixth tergites, and the Abd-B staining pattern in the CNS (see Figure 3 and Figure 4). The *iab-6^1^* and *iab-6^4^* show transformations of A6 to A5 for all phenotypes monitored. Meanwhile *iab-6^8^* shows only a partial transformation of A6 to A5 as seen by the sternite shape and trichome pattern on A6, which remain A6-like.

Previously, we identified a 2.8 kb element from *iab-6* that displayed the characteristics of an initiator in a transgenic reporter assay. Accordingly, this 2.8 kb fragment was shown to be able to drive the early expression of a *lacZ* reporter in a spatially restricted, pair-rule pattern, from PS11/A6 [22]. Unfortunately, as with other initiators, its precise function in the BX-C was never investigated genetically. We thus created a mutation that removes this 2.8 kb initiator fragment from the *iab-6* domain. The resulting mutant is named, *iab-6^1^* (see Figure 1B). According to the domain model, initiators should act as switches to turn on all of the enhancers in a domain. Thus, removal of the initiator from *iab-6* should result in a complete loss of *iab-6* function. This is, in fact, what we see. In flies homozygous for this deletion, A6 seems to be completely transformed into A5 (Figure 4A). This can be clearly seen on the adult cuticle where the 6^th^ sternite takes on the shape and bristle pattern characteristic of A5. The transformation can also be seen in the embryonic CNS, where the *Abd-B* expression pattern in PS11/A6 is replaced by the pattern normally found in PS10/A5 (Figure 4C).

In as much as the 2.8kb fragment had not been further dissected in the reporter gene assay, we decided to further narrow down the initiator by integrating smaller deletions from within the 2.8 kb fragment. In order to select these deletions, we first applied a bioinformatic approach (fly_Ahab: http://gaspard.bio.nyu.edu/fly_ahab.html) to identify regions in the *iab-6* domain that correspond to binding sites for known *Drosophila* transcription factors. Using this approach, we identified a potential *cis-*regulatory module in an ∼900 bp region of the *iab-6* initiator. This module contains the predicted binding sites for the Kruppel, Caudal and Hunchback proteins, and is supported by recent genome-wide ChIP analysis (http://genome.ucsc.edu). As previous work had indicated that initiators sense A-P position through the binding of the maternal, gap and pair-rule gene products [10], [12], [17], [20], [37]–[41], we decided to test if this ∼900 bp region is required for *iab-6* function in the BX-C. To do this, we created three overlapping deletions, *iab-6^2^*, *iab-6^3^*, and *iab-6^4^*, that each deletes this 900 bp region (Figure 1B). As flies homozygous for any of these three mutations display the same phenotype, we will concentrate on the smallest of these deletions, *iab-6^4^*, which deletes only a 927 bp sequence corresponding to the identified *cis*-regulatory module. Flies homozygous for *iab-6^4^* show a complete loss of *iab-6* function, like that seen in *iab-6^1^* flies. The extent of the transformation is corroborated by the tergite trichome pattern (Figure 4E) and the embryonic *Abd-B* expression pattern in the CNS, where the PS11/A6-specific pattern is replaced by the pattern normally found in PS10/A5 (Figure 4F). Based on these results, we conclude that this 927 bp fragment is absolutely necessary for *iab-6* activation of *Abd-B* in PS11/A6. The fact that a deletion of the initiator is capable of completely removing *iab-6* activity in the epidermis and the CNS, is consistent with the idea that the initiator functions as a switch to turn on all of the regulatory elements in a cis-regulatory domain. However, these results would also be consistent with the initiator being the sole positive regulatory element in A6/PS11. To rule out this possibility, we created another mutation that removes much of the *iab-6* cis-regulatory region but leaves intact the 2.8 kb initiator fragment. This mutation, called *iab-6^8^* (Figure 1), also shows a strong loss of *iab-6* function (Figure 4G). Thus, although the *iab-6* initiator is critical for *iab-6* function, it is not sufficient for *iab-6* activity.

One important point to note regarding *iab-6^8^* is that the loss-of-function (LOF) phenotype is slightly weaker than in *iab-6^4^*. The difference between these two mutants can be seen when examining the trichome pattern on the transformed A6. While the *iab-6^4^* mutation displays a transformed A6 with an A5-like trichome pattern (uniformly covered, Figure 4E), the *iab-6^8^* trichome pattern still resembles that of a wild-type A6 (Figure 4H). This suggests that although *iab-6^8^* removes most of the *iab-6* sequence, there is still some functionality left in the domain. This is an important finding because it supports a prediction of the domain model, which suggests that removal of cell-type specific enhancers would affect individual (or grouped) characteristic, while removal of initiator elements would affect all characteristics. To test this idea more directly, we next performed an initiator swapping experiment.

### Initiators act as domain control centers for *cis*-regulatory domains

The domain model suggests that initiators act as switches to turn on (or off) the various regulatory elements present in a domain. In the simplest state, this would mean that initiators would not participate directly in driving homeotic gene expression, but would simply coordinate the activity of cell/tissue-type specific enhancers along the A-P axis. If this were true, then we hypothesized that we should be able to transform a segment into another, simply by turning on the cell/tissue-type specific enhancers of domain in an area where they are normally off. Using our InSIRT method, we could do this by exchanging initiators from different domains.

For these experiments, we chose to remove the 927 bp *iab-6* initiator and replace it with the molecularly identified initiator from *iab-5*. The *iab-5* initiator is defined as a 1 kb DNA fragment (called IAB5) that, when cloned in front of the *Ubx-lacZ* reporter gene, activates strong β-galactosidase in a pair-rule fashion from PS10 [12]. Therefore, if the domain model is correct, by replacing the *iab-6* initiator with that of *iab-5*, we should be able to activate the enhancers required for PS11/A6 development in PS10/A5. As the difference in the expression pattern of *Abd-B* between PS10 and PS11 can be summarized as PS11 having a higher level of *Abd-B* expression than PS10 (and in more cells), we would expect ectopic activation of *iab-6* to be epistatic to the activity of *iab-5*. In other words, we expected to see a posterior transformation of A5 into A6.

**Figure 5:**
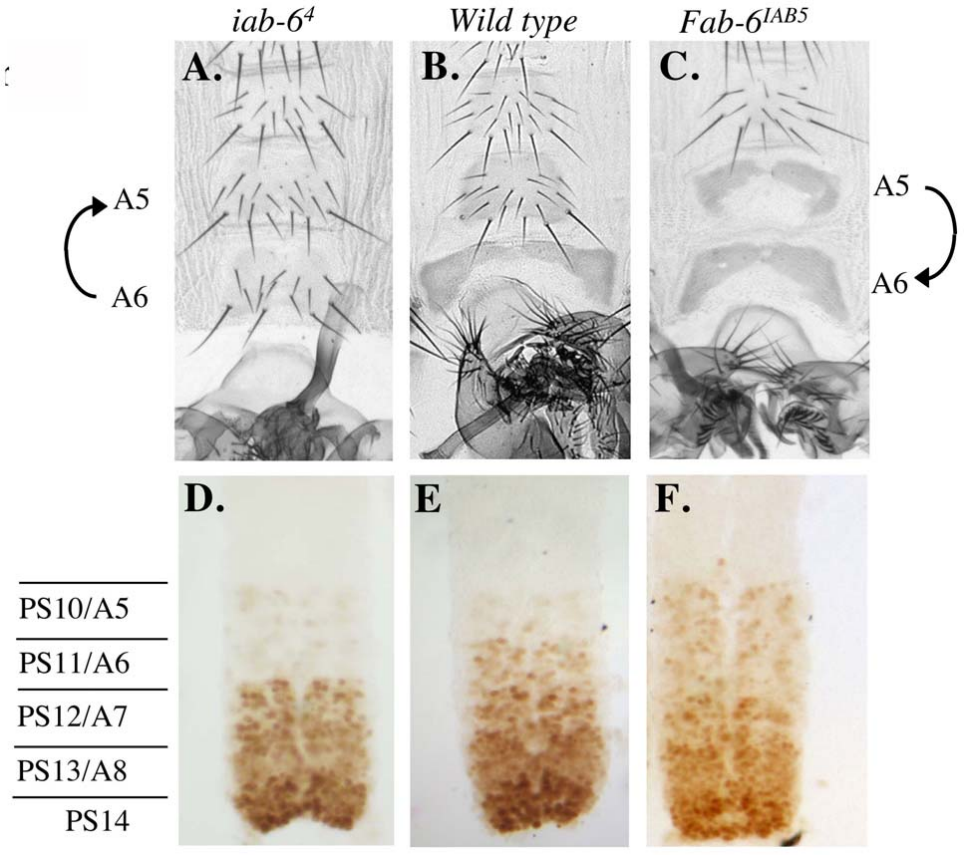
Phenotypes from initiator mutants. Genotypes are as follows: A. and D. *iab-6^4^*. B. and E. wild type. C. and F. *Fab-6^IAB5^*. A.–C. Show the ventral sternite cuticles made from adult males, homozygous for the genotype indicated above. Notice that A5 differs from A6 based on the sternite shape and the bristles present on the A5 sternite. The opposite homeotic transformations are highlighted by the direction of the arrows on the left and the right of the cuticles. D.–F. Show ventral nerve chords made from homozygous embryos of the genotypes indicated above. Parasegment borders are marked to the left.

As predicted by the domain model, the swapping of the *iab-6* initiator with that of *iab-5* results in a dominant A5 to A6 transformation that is stronger in homozygous flies. This type of posterior-directed abdominal transformation in the BX-C is typically called a *Frontabdominal* (*Fab*) transformation. For this reason, we have named this new mutation, *Fab-6^IAB5^*. Figure 5C shows an abdominal cuticle of a *Fab-6^IAB5^* homozygous male. In this fly, we observe an A6-like lack of A5 sternite bristles, an A6-like trichome pattern on the A5 tergite and a PS11/A6-like *Abd-B* pattern of expression in PS10/A5 (Figure 5F). The fact that A5 appears to be a copy of A6 suggests that everything required for the patterning of A6 is still present in the modified *iab-6* domain, but that these elements have simply been activated one segment too-anterior. These findings strongly support the model in which initiators function, not as enhancers to directly control homeotic gene expression, but rather as domain control centers to turn on the other *cis-*regulatory elements in a domain.

Interestingly, in the *Fab-6^IAB5^* mutation, A6 seems to be unaffected by the IAB5 swap. As mentioned above, the IAB5 fragment drives reporter gene expression in a pair-rule manner from PS10/A5 (i.e. not in PS11) [12]. In fact, reporter transgenes carrying the entire *iab-5* domain are still only capable of driving reporter gene expression in a pair-rule fashion [22]. If this assay reflects IAB5 activity *in vivo,* then what turns on *iab-6* in PS11/A6? Currently, we do not have a completely satisfying answer to this question. However, previous genetic studies tell us that the *iab-5 cis*-regulatory domain is indeed capable of working in PS11/A6. In *iab-6* mutants, for example, A6 is transformed into A5 [22], [42]. Thus, it seems clear that our interpretation of IAB5 activity from the transgenic reporter assay, oversimplifies IAB5 function. This is perhaps not too surprising, as the reporter gene assays were designed to simply test if a DNA fragment is capable acting as an enhancer. From our experiments, however, it seems that initiators may have a more complex function that is not reflected in the transgene assay.

### Mutations affecting *Fab-6* boundary function

During our dissection of the *iab-6* domain, we also created a number of deletions affecting *Fab-6* boundary function. Boundaries function to keep domains autonomous. Based on what was observed in other boundary deletions, we know that boundaries prevent both ectopic activation of posterior domains by elements present in anterior domains (initiators), as well as, prevent anterior domains from being silenced by posterior silencing elements (PREs) [18], [31]. The removal of a boundary element, therefore, results in a mixed transformation, where clones of cells in anterior segments become transformed towards cells of more-posterior segments, and clones of cells from posterior segments become transformed towards cells of more-anterior segments. Using our series of deletions, we have narrowed down the *Fab-6* boundary to an ∼650 bp region of the BX-C.

**Figure 6:**
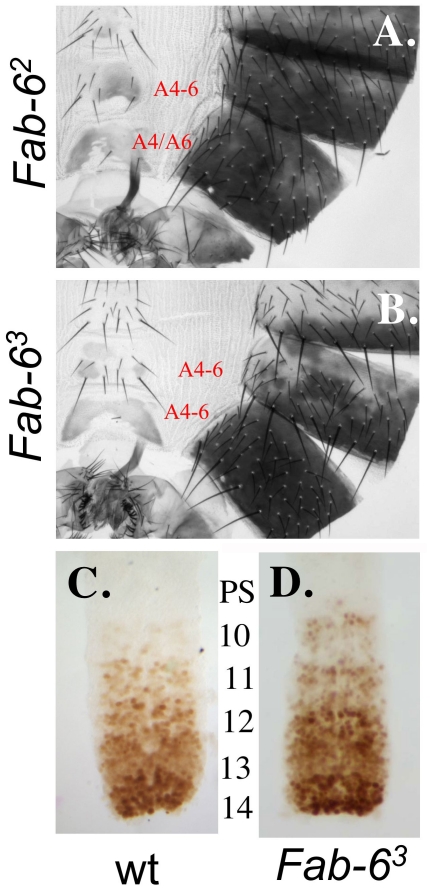
*Fab-6* boundary mutations. The genotypes of the adult male cuticles of A. *Fab-6^2^*, and B. *Fab-6^3^.* C. (wild type) and D. (*Fab-6^3^*) are embryonic nerve cords stained for Abd-B protein. Notice the increased level of Abd-B in PS10 in mutants (D.) relative to wild-type (C.).

Previous work from our lab [22] genetically mapped the *Fab-6* boundary to an ∼4.5 kb region of the BX-C between the distal (relative to the *Abd-B* transcription unit) breakpoints of the *iab-6^IH^* (3R:12712604) and *Fab-6,7^1^* (3R:12708067) mutations. By using deletions nested on the *Abd-B*-distal side of the *iab-5,6^CI^* mutation, we were able to quickly narrow down the location of the *Fab-6* boundary further. The first deletion we will speak about, *iab-5^Δ1^*, removes an ∼1.2 kb region from the distal side of *iab-5,6^CI^* but displays no visible phenotype. This indicates that neither the *iab-5* cuticle enhancer (see above), nor the *Fab-6* boundary element is removed by this deletion. On the other hand, two bigger deletions, removing ∼3.2 kb and ∼8 kb respectively (*Fab-6^1^* and *Fab-6^2^*), show mixed anteriorizing (LOF) and posteriorizing (GOF) transformations of A5 and A6 towards A4 or A6. An example of this (*Fab-6^2^*) can be seen in Figure 6A where we see a loss of bristles on the A5 sternite (indicative of a posterior-directed transformation of A5 to A6), and a loss of pigmentation on the A5 tergite (indicative of an anterior-directed transformation of A5 to A4). Meanwhile, in the A6 segment of each mutant, we see a gain of bristles on the sternite and a loss of pigmentation on the tergite, both indicative of an anterior-directed transformation (probably towards A4). Although very similar, we must note that the *Fab-6^2^* mutation displays a slightly stronger GOF phenotype than *Fab-6^1^* (data not shown). Consistent with this finding, a PRE has recently been mapped to the region differentiating the *Fab-6^1^* and *Fab-6^2^* mutations [43]. Thus, if *Fab-6^2^* functions like other boundary mutations, the enhanced *Fab-6* GOF phenotype is probably caused by deleting this silencing element and shifting the balance between GOF and LOF phenotypes.

The smallest mutation we created that displays an *Fab-6* phenotype is *Fab-6^3^* which deletes an ∼2 kb span of DNA between the proximal breakpoints of *iab-5^Δ1^* (3R:12706585) and *Fab-6^1^* (3R:12708661). As expected *Fab-6^3^* displays a phenotype similar to *Fab-6^1^* and *Fab-6^2^* (Figure 6B) with mixed gain- and loss-of-function phenotypes. Meanwhile, a deletion spanning the PRE region that differs between *Fab-6^1^* and *Fab-6^2^* shows no phenotype (*iab-6^Δ7^*, Figure 1), suggesting that the critical elements required for *Fab-6* function are all contained in *Fab-6^3^*. Thus, when combining our new data with those of the past, we can now narrow down the *Fab-6* boundary to an ∼650 bp region, spanning from the proximal breakpoint of *Fab-6,7^1^* (3R:12708067) to the distal breakpoint of *iab-6^Δ7^* (3R:12708714). Consistent with this mapping, it has recently been shown that this region contains binding sites for the insulator protein dCTCF [44], and that a fragment containing these dCTCF binding sites blocks enhancer-promoter interactions in an insulator reporter assay [45].

### Additional mutations in the *iab-6* region

Other deletions created for this study are presented here, solely for the purpose of completeness. These additional deletions are depicted in Figure 1 and their phenotypes are summarized in Table 1. Although we will not discuss these mutants in detail, we would like to point out two key issues surrounding these mutations. First, all deletions removing the initiator fragment show a loss of *iab-6* function comparable to that seen in *iab-6^4^*. This finding is in agreement with the domain model and our data, which suggests that the initiator is absolutely required for activating *iab-6* function in the cuticle and CNS. Second, we have also isolated a number of mutations that show no noticeable *iab-6* phenotype. These mutations, *iab-6^Δ7^*, *iab-6^Δ5^* and *iab-6^Δ6^*, remove a total of ∼9.8 kb of *iab-6* sequence without dramatically changing the morphology of the adult cuticle or modifying the *Abd-B* expression pattern in the embryonic CNS (data not shown). Does this mean that these sequences are without function? Absolutely not. In fact, we know that the region deleted in *iab-6^Δ7^* probably contains a PRE, whose deletion produces a phenotype when combined with the deletion of the *Fab-6* boundary. Also, as we have scanned only a small fraction of the possible developmental pathways in which *Abd-B* is involved, we believe that it is very likely that these other regions contain cell-type specific enhancers controlling *Abd-B* expression in other tissues than the CNS and the cuticle. Obviously, now that we have the ability to manipulate regions of the BX-C at a base-pair level, we now require equally precise methods to monitor potential phenotypic changes.

### The initiator and a hierarchical structure to gene regulation

For more than twenty years, much of the work on the BX-C has proceeded on the assumption that the BX-C cis-regulatory regions control homeotic gene expression through a multilayered, hierarchical process, summarized in the domain model. Key to this model was the idea that there existed specialized switch elements to control the activity state of the entire domain. Based on transgenic assays, these switch elements were thought to be special early embryonic enhancers, often called initiators. Although through the years, we, and others, have generated many results consistent with this model, we were never able to directly test initiator function *in situ*, due to experimental difficulties. Thus, a key prediction of the domain model went untested for decades. Here, we have finally provided the data confirming the key role of the initiator in domain activation.

Besides being important for studies on BX-C gene regulation, our findings highlight the possibility of having elements whose sole function may be to control the activity state of other elements. Although we still do not understand how this is accomplished mechanistically, we believe that it is probably through modifying the local chromatin environment around the enhancers. In fact, taking into account the enhancer activity of initiators in transgenic constructs, we are left with an intriguing and testable model for initiator action. As mentioned above, initiators were first isolated as early embryonic enhancers that turned on reporter gene expression in an A-P restricted manner. It has been known for years that the *cis*-regulatory sequences of the BX-C are transcribed in a parasegment-specific manner where transcripts from each *cis*-regulatory domain are expressed along the A-P axis in correspondence to where a domain is expected to be active [46]–[49]. In other experiments it has been shown that forced transcription across PREs in the BX-C can prevent Pc-dependent silencing, and hence, activate a domain [50]–[52]. Thus, by combining these findings, it is possible to imagine a causal relationship between the initiator and transcription, and the transcription of a domain and domain activation. Accordingly, we propose that initiators might act as enhancers, responding to gap and pair-rule gene products to activate transcription from promoters within the *cis*-regulatory domains. In doing so, they would indirectly activate homeotic gene expression by preventing the *Pc* silencing of homeotic gene enhancers. Using the tools developed here, we are now in the process of testing this model.

Another question that we can now address using InSIRT is whether or not initiators are required later in development. Thus far, we have been discussing initiators as only functioning early in development. It is still possible, however, that initiators are constantly required for domain activation or that they play a later role in the regulation of homeotic gene expression. Although the initiator being constantly required to keep a domain active cannot be ruled out, based on our current understanding of initiator function, we do not believe this to be the case. Perhaps the strongest evidence supporting this belief comes from transgenic assays. In transgenic assays, initiator fragments seem to respond to the maternal, gap and pair-rule gene products. Upon disappearance of the early expression pattern of these proteins, initiator activity in transgenes often disappears, or produces a pattern of expression not restricted along the A-P axis (if not paired with a PRE/maintenance element). Based on this, we believe that their role in coordinating the activity of a domain is probably limited to early development. However, this does not mean that all initiators would have no activity outside of initiation phase, only that their function in domain initiation would be limited to early embryogenesis. As mentioned above, the pattern of reporter gene expression driven by some initiator fragments degenerates into a cell-type specific enhancer-like pattern later in development. Therefore, it may be possible that DNA fragments with initiator function may also contain cell-type specific enhancers.

### InSIRT as a method to study gene expression

The transgenic reporter assay has played an important role in shaping our understanding of eukaryotic gene expression [53]. Its advantages stem from its speed and the cleanliness of the approach in isolating *cis*-regulatory elements away from competing or obfuscating signals. To gain these advantages, reporter assays must make a number of critical assumptions. First, they must assume that an activity performed by an element in the reporter assay, is the activity performed by the element *in vivo*. Second, they must assume that critical transcriptional activities can be tested using the molecular construct devised. And third, they must assume that it is through the addition of these *cis*-regulatory activities that controlled gene expression is achieved. However, these assumptions are not always correct. Although in the study of the BX-C, transgenes have been extremely useful in estimating the activity of elements, our work on the initiator and our previous work on boundary elements [54] highlight how sometimes the activity seen in transgenes assays only represents a portion of an element's activity *in vivo*.

InSIRT is a complementary approach. Relative to the transgenic approach, InSIRT has one main advantage: it tests for changes on biologically relevant targets without necessarily simplifying or assuming an activity. This is key when trying to understand unusual regulatory elements, like initiators. Furthermore, this advantage can be achieved with only a small penalty in time, as, once the homologous recombination has been performed, InSIRT mutagenesis takes only as much time as establishing a single transgenic line. Because transgenic approaches often require the analysis of multiple lines to control for genomic position effects, this penalty is further reduced. Thus, we believe that InSIRT offers scientists a powerful new tool that can be used in combination with classical transgenic methods to better study gene regulation.

## Materials and Methods

### Fly methods and phenotypes

All crosses, and cuticle preparations were performed using standard *Drosophila* methods. *Abd-B* antibody staining was performed as in [22]. *Abd-B* monoclonal antibody was purchased from the “Developmental Studies Hybridoma Bank” at the University of Iowa. Injection experiments were performed using cleaned DNA preparations (Qiagen) and injected into the *iab-5,6^CI^* flies stocks containing an X chromosome expressing the φC31 integrase enzyme under the control of the *vasa* promoter [36] see http://www.frontiers-in-genetics.org/flyc31/).

Phenotypes depicted are representative of the genotypes shown. As some of the boundary phenotypes seem to be clonal in nature, there is an occasional variance in the exact number of bristles and the exact pattern of trichomes. We have, therefore, attempted to choose an average representative cuticle for display. Otherwise, the phenotypes can be considered 100% penetrant.

### Homologous recombination

Creation of a donor vector for homologous recombination: An *AscI-NotI* fragment containing the *yellow* reporter gene flanked by the two *loxP* sites, and a 255 bp *attP* integration site was cloned into pW25 digested with *AscI-NotI* to create the pY25 plasmid. Homology regions of ∼4 kb were created by PCR using the following primer pairs: IAB7-*AvrII*: CCTAGGCGGCGAACAGTAGGGAAG and Fab7-*AscI*: CAGC-AAAAATCGTAAAAAAG, and IAB5-*NotI*: GCGGCCGCGGTCAGTAAACGG-GTCCC and IAB5-SpHI: GCATGCACTGGCGACATTTCTC. These homology regions were then cloned into the pY25 vector in the *AvrII* and *AscI* sites or the *NotI* and *SphI* sites respectively. The resulting P-element vector, Py-del *iab-6*, was injected into *yw* flies and transformants were isolated as *yellow* ^+^ flies. Homologous recombination was performed using two independent transformants and the ends-out homologous recombination method of Gong and Golic [34].

Potential homologous recombinants were isolated based on their yellow pigmentation limited to the segments posterior and including A5 (as a result of being in *iab-5*). Genomic southern blots, however showed that all identified events were aborted recombination events in the BX-C. As aborted events happened on each side of the targeted region, we were able to generate the final “planned” deletion by recombining two chromosomes each having recombined properly at one homology region. This recombination was mediated by the Cre recombinase at the *loxP* sites left behind after removal of the *yellow* reporter gene. The final chromosome, *iab-5,6^CI^*, was verified by genomic southern blot and sequencing.

### Generation of integration vectors

A base vector containing the 19.3 kb area deleted in the *iab-5,6^CI^* deletion, a 288 bp *attB* sequence, a yellow reporter, and one *loxP* site (called KsY-iab6H) was generated using gap-repair recombineering ([55], [56] see Figure 1B). For this, ∼500 bp PCR products were generated to both the *iab-5* and *iab-6* regions (each starting at the breakpoints of the *iab-5,6^CI^* deletion) using the following primers:

Iab-5 N: ATAAGAATGCGGCCGCGGTGCGTTTCCATTT-TCCCTAGG

Iab-5 new (+PmeI): CTCACCATAGAGCACCACGTTTAAACGTCGT-CCGGAAATGGCAACCAG

Iab-6: CTTTGCCAGCTTTTGCCACTCGTCC

Iab-6P(+PmeI): GGTTGCCATTTCCGGACGACGTTTAAACGGTG-AAGGCGCG-AAACTGTGA.

These two products were linked using overlap PCR. This 1 kb fragment was then cloned into a vector containing a 288 bp *attB* sequence, a yellow reporter, and one *loxP* site (in that order), resulting in the plasmid, Ks-Y *attB*-*loxP*.

The Ks-Y *attB*-*loxP* plasmid was digested with *PmeI* and used in recombineering experiments to capture the 19.3 kb region deleted in the *iab-5,6^CI^* deletion. All recombineering procedures were performed as in ([55], [56]. Basically, the digested plasmid was transformed by electroporation into heat-shock induced competent cells of the EL350 recombineering bacterial strain that was previously transformed with the *Abd-B* region-containing BAC, BACR24L1 (BACR24L18, GenBank: AC095018). The resulting plasmid is called KsY-iab6H.

The KsY-iab6H was then modified using recombineering. For the initiator deletions, we generated PCR fragments containing a Kanamycin selector gene surrounded by two FRT sites, using primers containing 5′ leaders with 50 bp of homology to the region flanking the sequences to be deleted. The FRT-kanamycin-FRT DNA template for PCR came from pGEM1-K7-FRT-Kan-FRT (kindly provided by François Spitz). For the modification of KsY-iab6H by recombineering, PCR targeting fragments (containing an FRT-Kan-FRT cassette) were generated using the primers listed in Table 2.

For the recombineering of the *iab-6/iab-5* initiator swap, the *iab-5* initiator was amplified by PCR using the following primers:

s-sub-iab5 5′ATGGCGCGCCGGAGGCGGCAAATGCACAAAG3′

as-sub-iab5 5′ATGGCGCGCCTACTACGCCGATTCTGCTGG3′

Two fragments (of 1.7 kb and 1.3 kb, respectively), each homologous to one side of the *iab-6* initiator were amplified by PCR using the following primers:

sub1-AscI: 5′ATGGCGCGCCCAGATTTCTGGAATGGTTAGAAAAT-ATTAAAGG3′

sub2-NotI: 5′ACTCGCGGCCGCTCGGAAACATCAAAGCATCAGCA-AC3′,

sub3-AscI: 5′ATGGCGCGCCGCAGTAAGTTAATATATTTTATAC-TCC3′

sub4-NotI: 5′ACTCGCGGCCGCAGAGAAATATATTCTTTGGCAG-CGAGC3′.

The IAB5 initiator was cloned between these regions of homology using the AscI restriction site to create the vector, Target iab5(+). An FRT-Kan-FRT cassette was amplified by PCR using the following primers:

5′ SnaBI: 5′ACATGGAAAACAACAGTTTCAATCAGGTCATGTAC-CTAATAAATGTATACGAATACAAGCTTGGGCTGCAGG3′

3′ SnaBI 5′AGCTTACATTTTGATAGCTTAAGTGGATGTTTCAAGGA-ATTTATATATACCTCGCCCGGGGATCCTCTAGAG3′

This cassette was then cloned into a unique SnaBI site within the 1.7 kb homology domain (245 bp from the IAB5 initiator) of Target iab5(+), to make Target iab5(+) Kan FRT. A NotI fragment containing the two homology regions, the IAB5 initiator and the Kan-FRT cassette was then used to recombineer the *iab-5* swap integration vector. The recombineering was otherwise performed as above.

Upon recombineering on the KsI-iab6H plasmid, the plasmids carrying the designed deletion were selected on Kan plates. The Kanamycin cassette was then removed using a bacterial strain expressing the flipase enzyme under an inducible arabinose promoter (EL 250; [55].
